# Granulocyte colony-stimulating factor potentiates all-*trans* retinoic acid-induced granulocytic differentiation in acute promyelocytic leukemia cell line HT93A

**DOI:** 10.1186/s12935-015-0176-2

**Published:** 2015-03-12

**Authors:** Yoshihito Uchino, Noriyoshi Iriyama, Yoshihiro Hatta, Masami Takei

**Affiliations:** Division of Hematology and Rheumatology, Department of Medicine, Nihon University School of Medicine, 30-1 Oyaguchi Kami-cho, Itabashi-ku, Tokyo 173-8610 Japan

**Keywords:** Granulocyte colony-stimulating factor, All-*trans* retinoic acid, Differentiation, Signal transducer and activator of transcription, Ruxolitinib, HT93A

## Abstract

**Background:**

Granulocyte colony-stimulating factor (G-CSF) promotes proliferation, survival, and differentiation of myeloid-linage leukemic cells, as well as normal hematopoietic cells. Terminal granulocytic differentiation can be induced in acute promyelocytic (APL) cell line HT93A by G-CSF and all-*trans* retinoic acid (ATRA). Because the detailed mechanism has never been shown, we investigated the signal transduction pathway in granulocytic differentiation by G-CSF, alone or in combination with ATRA.

**Methods:**

HT93A cell viability and growth were investigated by trypan blue exclusion assay. Cell differentiation was assessed by CD11b and CD34 expressions. Intracellular protein expressions were also evaluated by flow cytometry after fixation and permeabilization.

**Results:**

ATRA (100 nM) induced granulocytic differentiation (upregulation of CD11b and downregulation of CD34) and the effect was potentiated by addition of G-CSF, while G-CSF alone had no effect on HT93A cells. The addition of G-CSF to ATRA had little or no effect on NB4 and THP-1 cells in comparison to ATRA alone. G-CSF receptor expression was reduced by ATRA treatment in a time-dependent manner. After 5 days’ incubation with ATRA, the expression levels of signal transducer and activator of transcription (STAT) 3, and phosphorylated STAT3 and STAT5, were significantly reduced. STAT5 was strongly activated by G-CSF stimulation in ATRA-pretreated cells in comparison to untreated cells. In contrast, STAT3 showed no response to G-CSF. Janus kinase (JAK) inhibitor ruxolitinib (320 nM) had little or no effect on ATRA-induced differentiation, but eliminated the enhancing effect of G-CSF, as evidenced by the levels of CD11b and CD34 expression. These results suggest G-CSF activates STAT5 through the JAK pathway in combination with ATRA, resulting in myeloid differentiation in HT93A cells.

**Conclusions:**

In conclusion, activation of the JAK-STAT pathway is likely essential for inducting differentiation in the APL cell line HT93A; thus, monitoring its expression and activation is important for predicting clinical efficacy and understanding the mechanisms of cytokine-dependent myelopoiesis, proliferation, and differentiation of acute myeloid leukemia.

## Background

Granulocyte colony-stimulating factor (G-CSF) is a myeloid growth factor that promotes proliferation, survival, and differentiation of myeloid-lineage leukemic cells, as well as normal hematopoietic cells [1,2]. Clinical studies have revealed that G-CSF is an effective promoter of granulocyte recovery [3]. G-GSF has also been used for its priming effect, enhancing the sensitivity of leukemia progenitor cells to cytotoxic agents with or without all-*trans* retinoic acid (ATRA) to improve therapeutic outcomes in acute myeloid leukemia (AML) [4-7].

Studies in various cell lines have revealed that G-CSF functions through the Janus kinase (JAK)-signal transducer and activator of transcription (STAT) pathway [8-10]. STAT3 and STAT5 are the major factors that would be affected by G-CSF treatment [11-13]. Although basic studies of G-CSF in various *in vitro* systems have been performed [8-17], the efficacy and mechanisms of G-CSF, alone or in combination with ATRA, are not well understood in the context of differentiation induction.

Acute promyelocytic leukemia (APL) is characterized by a balanced reciprocal translocation between chromosome 15 and 17, resulting in fusion of the promyelocytic leukemia and retinoic acid receptor alpha genes [18-20]. ATRA has become the first-line treatment for patients with APL due to its specific effect on differentiation induction and high complete remission rate [21,22]. Established by Kishi et al., APL cell line HT93A carries t(15;17), is sensitive to ATRA, and its differentiation is enhanced by treatment with G-CSF [23,24].

In this study, we examined the differentiation mechanism of ATRA and G-CSF, alone or in combination, in HT93A cells. Our data suggest enhanced differentiation induction through the JAK-STAT pathway is induced by combination treatment with ATRA and G-CSF in AML.

## Results

### Differentiation of HT93A cells by ATRA and G-CSF

**Figure 1:**
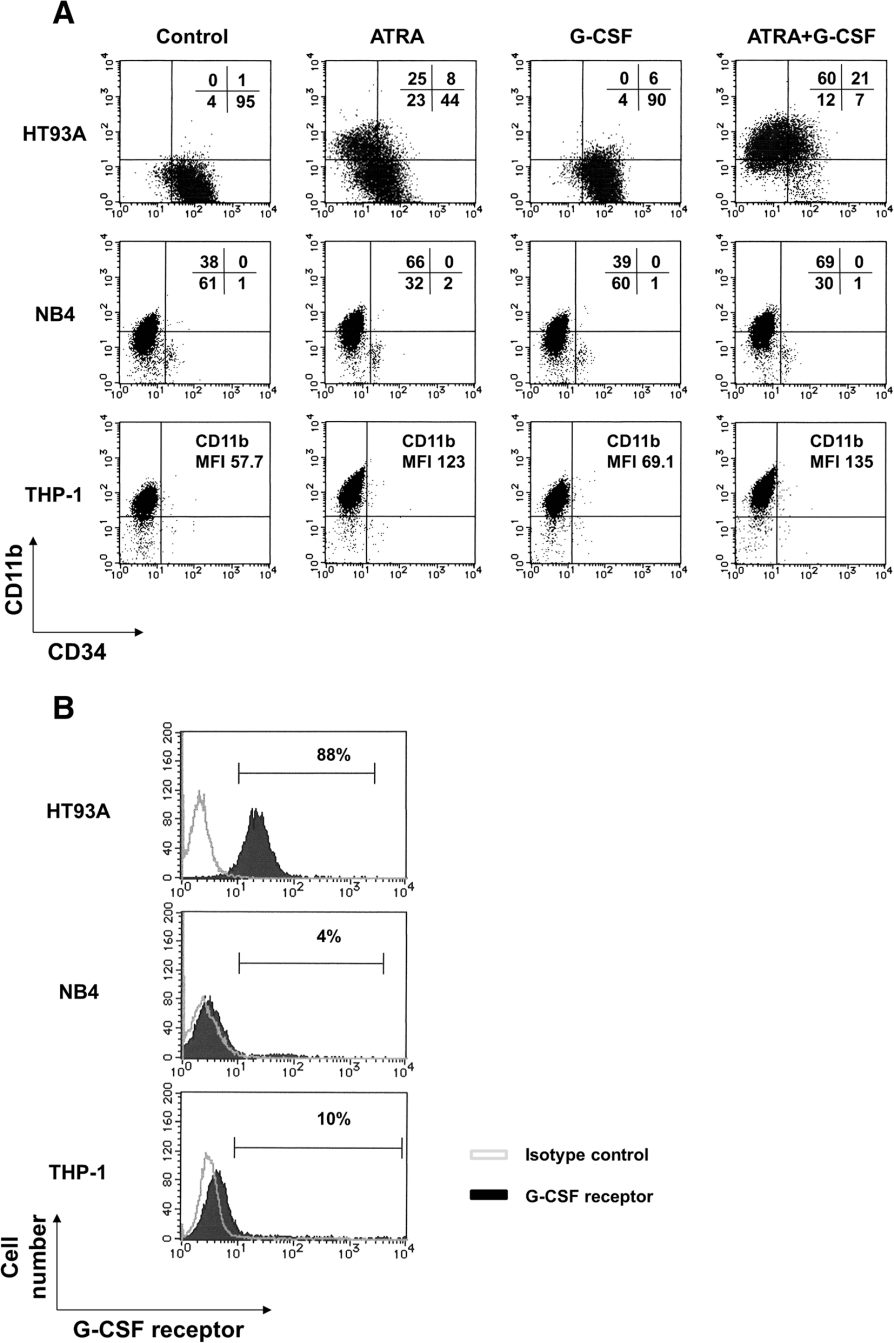
**Granulocytic differentiation and G-CSF receptor expression.** Differentiation profile of surface antigen expression in HT93A, NB4 and THP-1 cells **(A).** These cell lines were treated with 100 nM ATRA and 50 ng/mL G-CSF, alone or in combination, for 7 days (HT93A) and 48 h (NB4 and THP-1). Because THP-1 cells express high levels of CD11b in the control, CD11b expression is reported as the mean fluorescence intensity (MFI). Expression of G-CSF receptor in HT93A, NB4, and THP-1 cells **(B)**. HT93A specifically exhibited ATRA-induced CD11b expression and a decline in CD34, with strong G-CSF receptor expression. Expression profiles of CD11b, CD34, and G-CSF receptor were evaluated by flow cytometry. Experiments were performed twice or three times, each yielding similar results.

After treatment with 100 nM ATRA for 7 days, expression of major differentiation marker CD11b increased, while primitive marker CD34 decreased in HT93A cells (Figure 1A). Treatment with G-CSF alone did not affect these markers; however, ATRA plus G-CSF potentiated CD11b upregulation and CD34 downregulation in comparison to ATRA alone. Differentiation in NB4 and THP-1 cells was induced by ATRA; however, G-CSF alone or in combination with ATRA had little or no effect on these cell lines. HT93A alone showed high levels of CD34 and G-CSF receptor expression (Figure 1A and B).

### G-CSF activates STAT5 rather than STAT3 in the presence of ATRA

**Figure 2:**
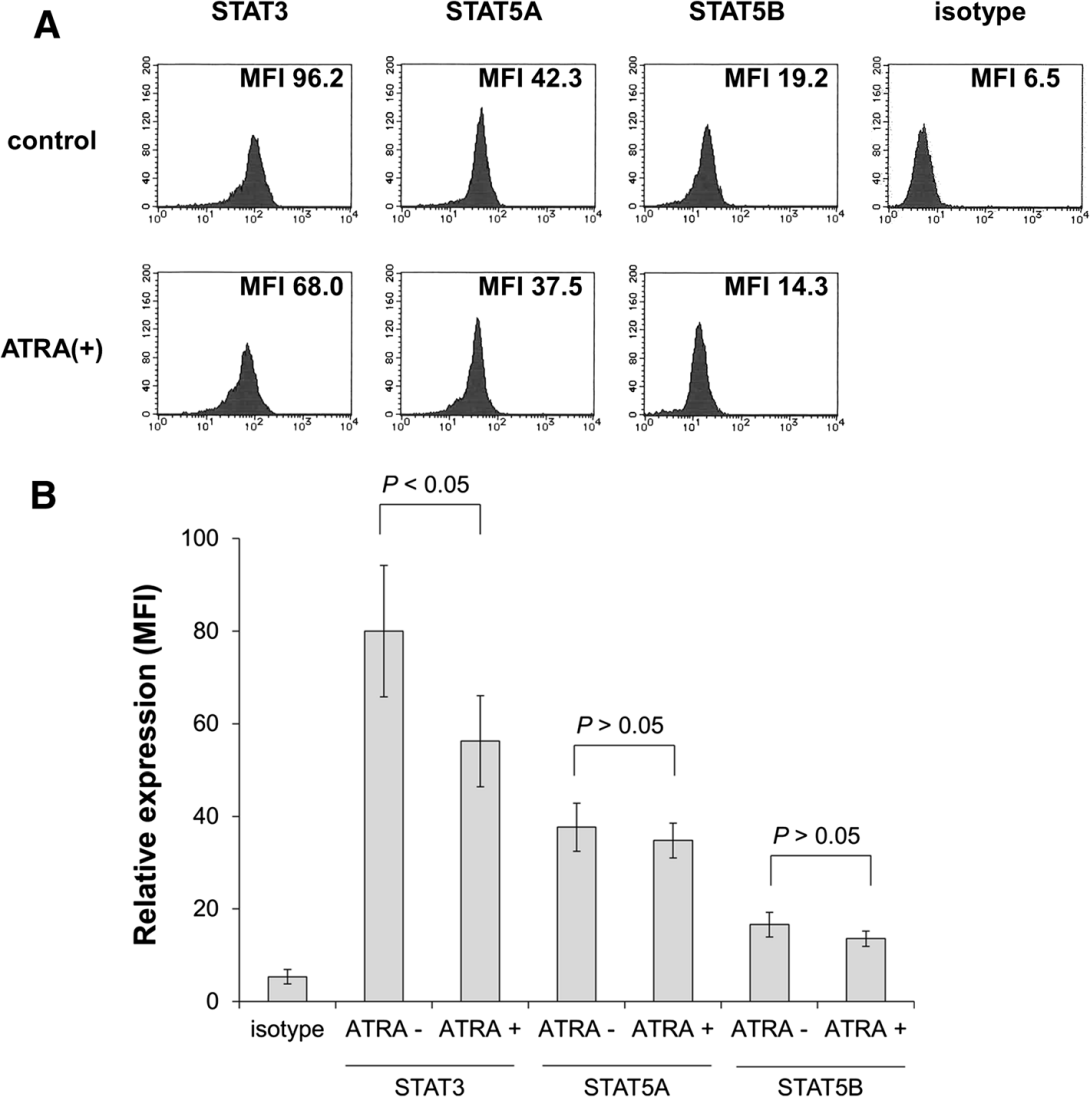
**Flow cytometry to assess expression of STATs in HT93A cells.** After treatment with or without 100 nM ATRA for 5 days, STAT3, STAT5A, and STAT5B expression was evaluated. Histograms **(A)** and bar graphs **(B)** represent STAT3, STAT5A, and STAT5B expression in the presence or absence of ATRA. Experiments were independently repeated three times and results are shown as mean ± SD. *NS* not significant.

**Figure 3:**
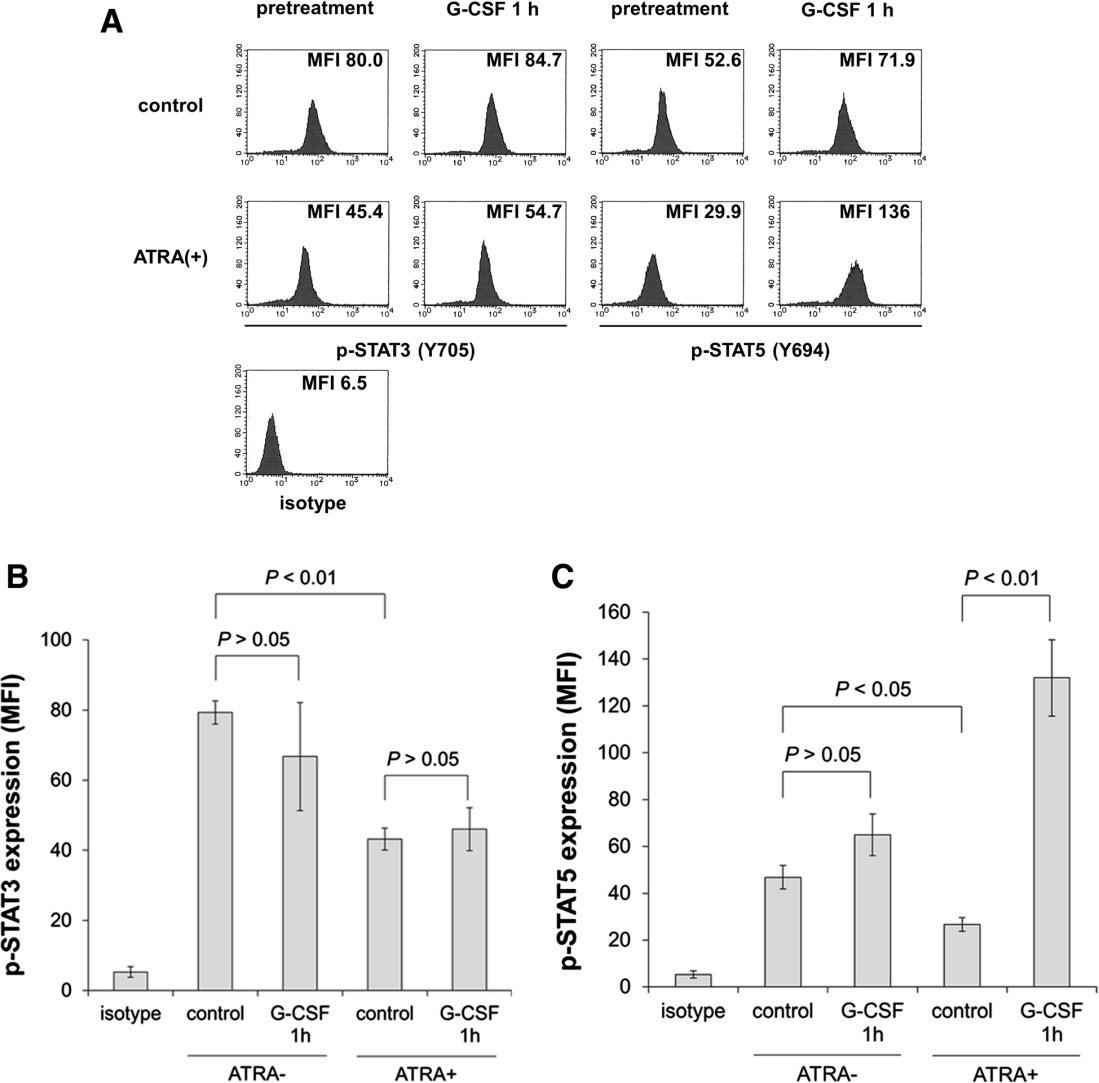
**Flow cytometry analysis of phosphorylated STAT expression in HT93A cells.** After treatment with or without 100 nM ATRA for 5 days, expression of phosphorylated STATs was evaluated before or after 50 ng/mL G-CSF stimulus for 60 min. Histograms **(A)** and bar graphs represent phosphorylated STAT3 **(B)** and STAT5 **(C)** expression. Experiments were independently repeated three times and results are shown as mean ± SD. *NS* not significant.

After treatment with 100 nM ATRA for 5 days, the expression levels of STAT3, STAT5A, STAT5B, and phosphorylated STAT3 and STAT5 were evaluated by intracellular staining followed by flow cytometry as described in the Materials and methods. ATRA alone inhibited expression of STAT3 (Figure 2A and B). Furthermore, phosphorylated STAT3 and STAT5 were also reduced (Figure 3A-C). After stimulation with 50 ng/mL G-CSF for 60 min, STAT5 phosphorylation was observed in ATRA-treated cells, although the response was not significant in untreated cells (Figure 3A and C). In contrast, STAT3 phosphorylation was unaltered by G-CSF stimulation in untreated and ATRA-treated cells (Figure 3A and B).

### Growth inhibition by Jak inhibitor ruxolitinib

**Figure 4:**
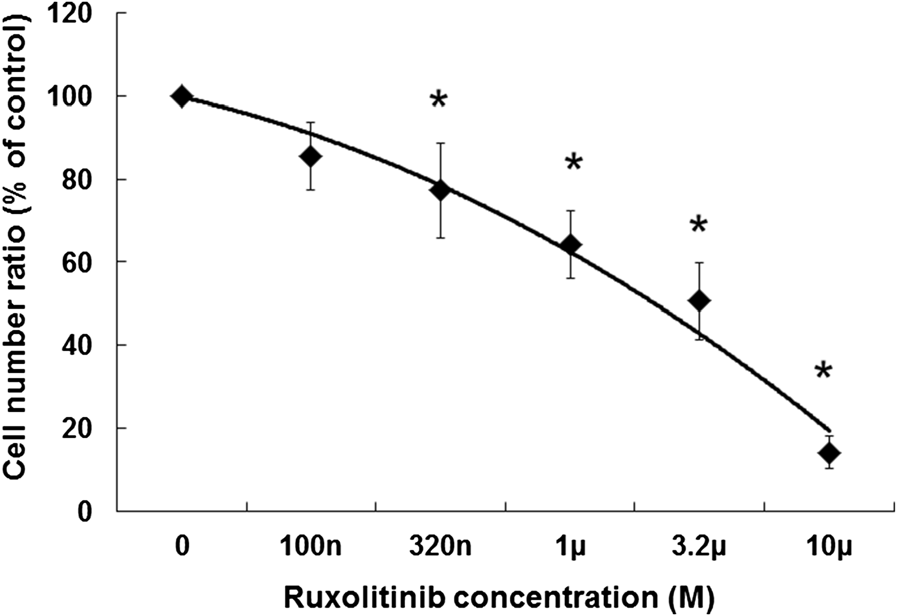
**Growth inhibition by ruxolitinib in HT93A cells.** After treatment for 7 days, growth inhibition was determined by trypan blue exclusion assay. Ruxolitinib inhibits cell growth in a dose-dependent manner. More than 320 nM ruxolitinib significantly inhibited growth. Experiments were independently repeated three times and results are shown as mean ± SD. *p < 0.05 vs. control.

Differentiation induction by G-CSF likely occurs via the JAK-STAT signaling pathway; therefore, we investigated whether JAK is associated with G-CSF-induced granulocytic differentiation in HT93A cells. We applied the JAK inhibitor ruxolitinib, which is used to treat myelofibrosis [25]. Growth inhibition was assessed in order to determine the optimal concentration of ruxolitinib. After dose titration of ruxolitinib for 7 days, cell growth was evaluated by trypan blue exclusion. HT93A cells were sensitive to ruxolitinib, which inhibited growth in a dose-dependent manner, with a significant difference in the concentration of more than 320 nM compared to control (Figure 4). Thus, we chose to use 320 nM ruxolitinib for subsequent experiments.

### Ruxolitinib inhibits STAT5 activation by G-CSF in the presence of ATRA

**Figure 5:**
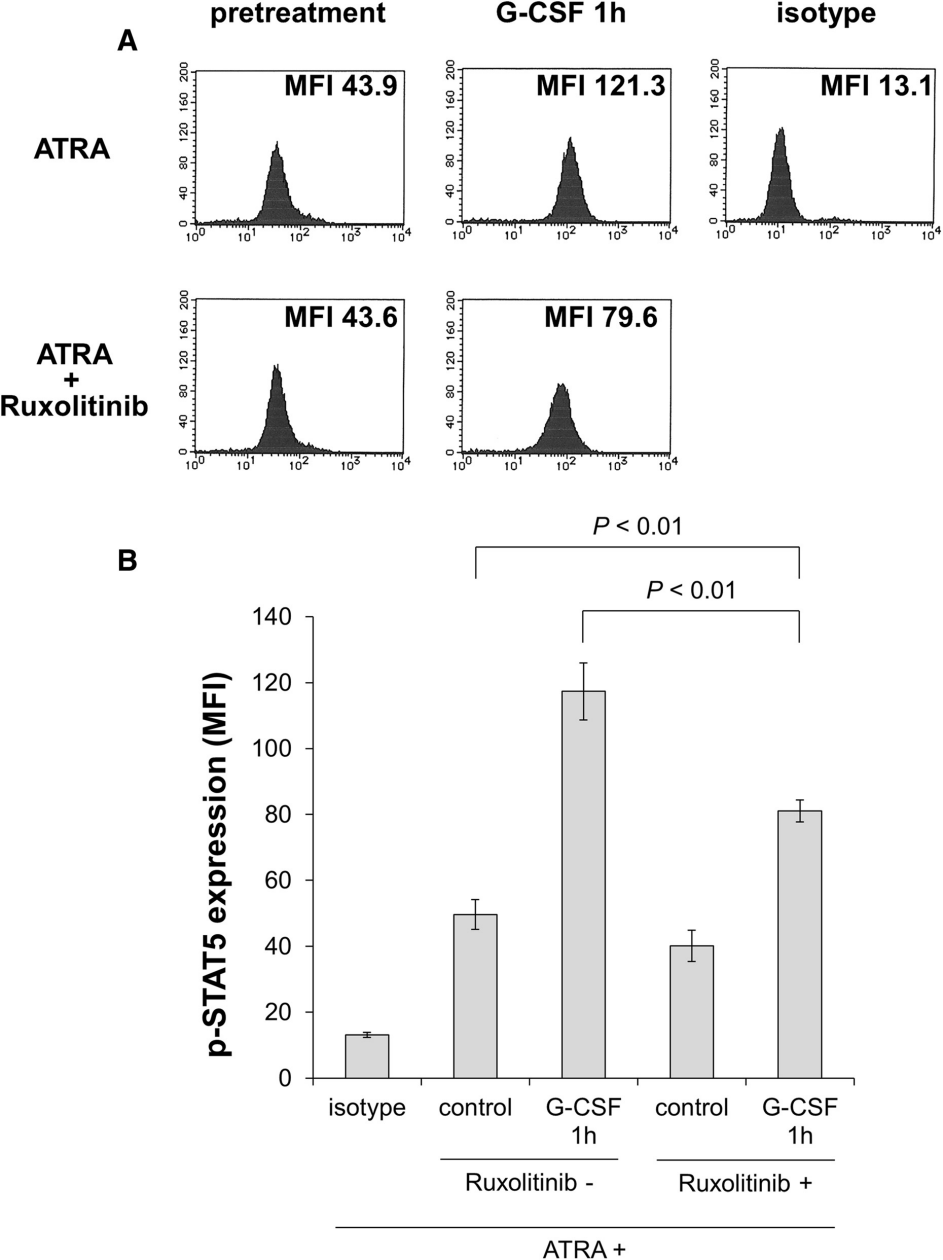
**After treatment with 100 nM ATRA with or without 320 nM ruxolitinib for 5 days, HT93A cells were stimulated with 50 ng/mL G-CSF for 60 min.** Expression of phosphorylated STAT5 was evaluated by intracellular staining and analyzed by flow cytometry. Ruxolitinib-treated HT93A cells showed significantly lower phosphorylated STAT5 expression. Histograms **(A)** and bar graphs **(B)** represent phosphorylated STAT5 expression. Experiments were independently repeated three times and results are shown as mean ± SD.

We assumed that the potentiation effect of G-CSF occurs via the JAK-STAT5 pathway in the presence of ATRA; thus, we sought to determine whether the JAK inhibitor ruxolitinib inhibits G-CSF-induced STAT5 activation. After treatment with 100 nM ATRA with or without 320 nM ruxolitinib for 5 days, HT93A cells were stimulated with 50 ng/mL G-CSF for 60 min. The expression levels of phosphorylated STAT5 were evaluated by intracellular staining followed by flow cytometry. As shown in Figure 5A and B, HT93A cells treated with ATRA plus ruxolitinib showed significantly lower expression level of phosphorylated STAT5 in comparison to those treated with ATRA alone.

### G-CSF-induced differentiation is abrogated by addition of ruxolitinib

**Figure 6:**
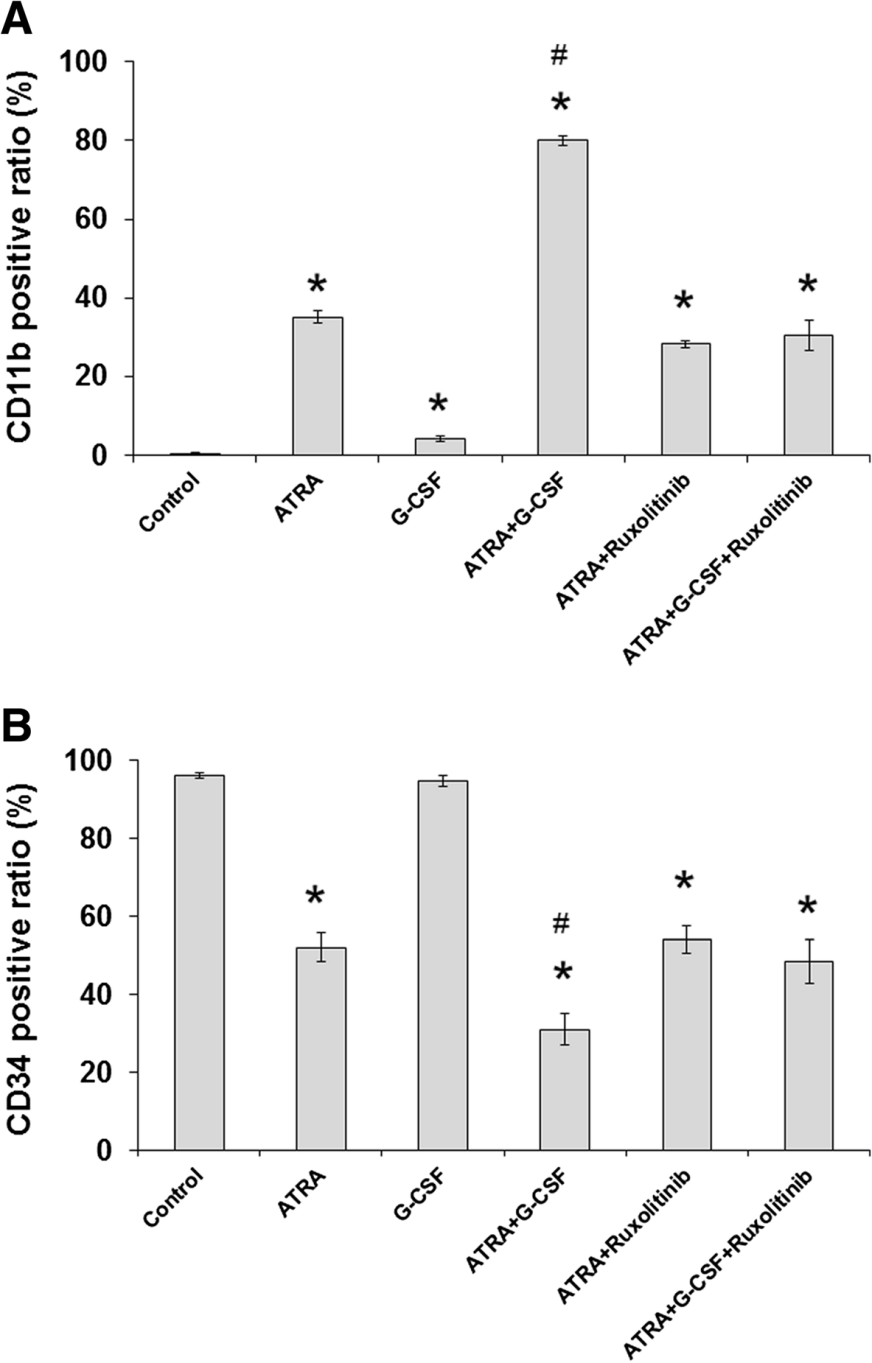
**Abrogation of G-CSF-induced differentiation with ruxolitinib.** HT93A cells were treated with 100 nM ATRA, with or without 50 ng/mL G-CSF, in the presence or absence of ruxolitinib for 7 days. CD11b **(A)** and CD34 **(B)** expression are shown. Ruxolitinib had little or no effect on ATRA-induced alterations of CD11b and CD34, but abrogated the enhancing effect of G-CSF. Experiments were independently repeated three times and results are shown as mean ± SD. *p < 0.05 vs. control; #p < 0.05 vs. ATRA.

We next investigated the effect of ruxolitinib with ATRA alone or in combination with G-CSF on cell differentiation. HT93A cells were treated with 100 nM ATRA, with or without 50 ng/mL G-CSF, in the presence or absence of ruxolitinib for 7 days. ATRA in combination with G-CSF induced CD11b expression and inhibited CD34 expression in comparison to treatment with ATRA alone (Figure 6A and B). While combination with ruxolitinib had little effect on ATRA-induced differentiation, enhancement of ATRA by G-CSF was almost completely abrogated by ruxolitinib, as shown by changes in CD11b and CD34 expression (Figure 6A and B).

### G-CSF receptor is downregulated by ATRA

**Figure 7:**
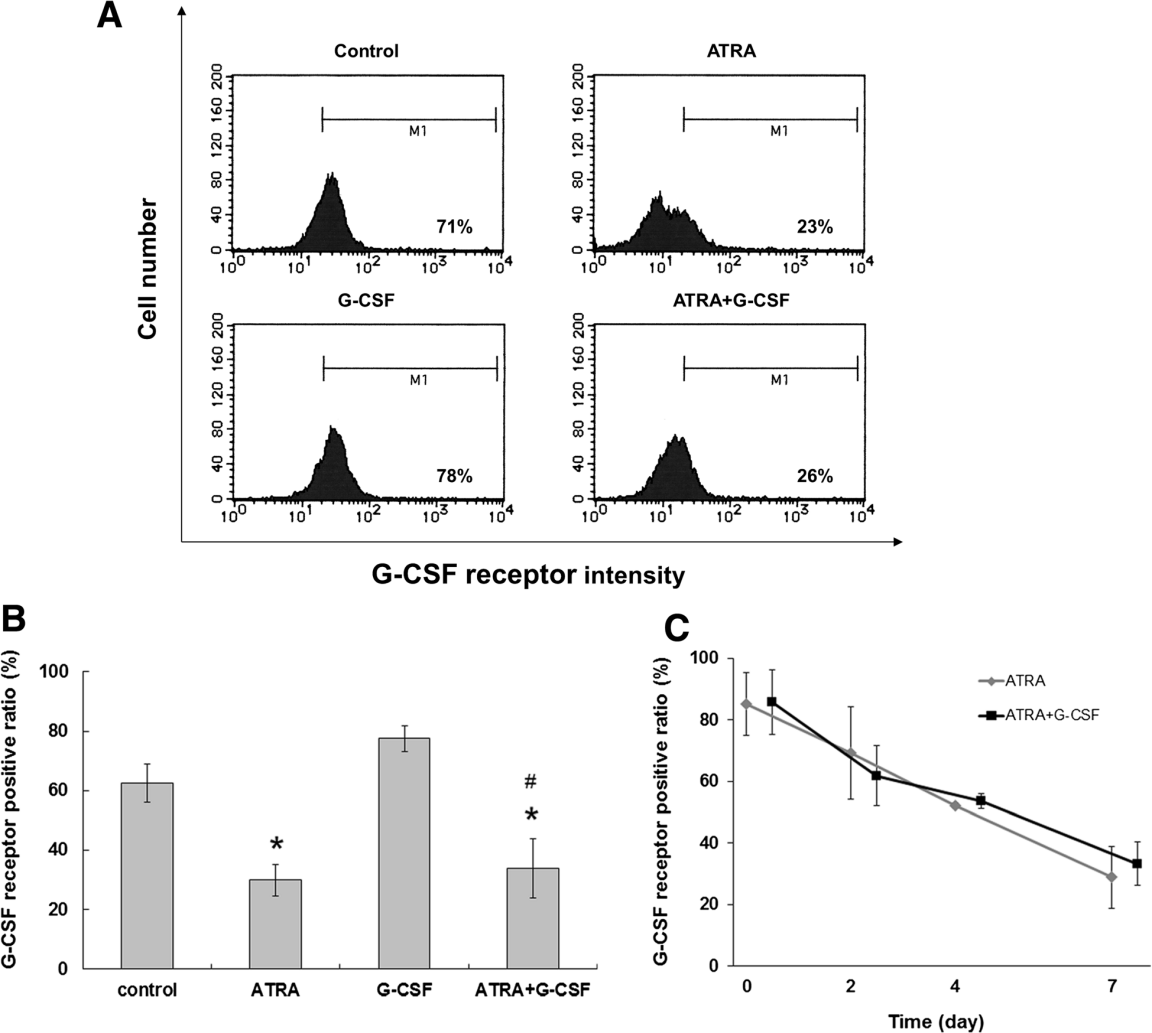
**Alteration of G-CSF receptor expression with ATRA.** HT93A cells were treated with 100 nM ATRA with or without 50 ng/mL G-CSF for 7 days. (**A** and **B**) G-CSF receptor expression was significantly downregulated by ATRA regardless of the presence or absence of G-CSF. G-CSF alone had no effect on G-CSF receptor expression. **(C)** The alteration of G-CSF receptor was time-dependent. Experiments were independently repeated three times and results are shown as mean ± SD. *p < 0.05 vs. control; #p < 0.05 vs. ATRA.

HT93A cells were treated with 100 nM ATRA with or without 50 ng/mL G-CSF for 7 days and expression levels of G-CSF receptor on the cell surface were investigated using flow cytometry. G-CSF receptor expression was significantly downregulated by ATRA, regardless of the presence or absence of G-CSF (Figure 7A and B); expression of the G-CSF receptor was repressed in a time-dependent manner (Figure 7C). G-CSF alone had no effect on G-CSF receptor expression (Figure 7A and B).

## Discussion

Our study demonstrated that G-CSF potentiates ATRA-induced granulocytic differentiation by enhancing STAT5 activation through the JAK/STAT pathway in HT93A cells. The JAK-STAT pathway, especially JAK1/2 and STAT3/5, is essential to the mechanism of G-CSF efficacy [11-13,26,27]. We showed that STAT5 activation by G-CSF was apparent only when G-CSF was combined with ATRA. We also found that STAT5 activation was associated with cell differentiation, as shown by the altered expression of surface antigens in HT93A cells. Although STAT3 activation by G-CSF has been well studied in various leukemia cell lines, we did not observe it here in HT93A cells. While most prior studies of G-CSF have focused on the activation of STAT3 or STAT5 in leukemia cell lines, Tanaka et al. [28] demonstrated that G-CSF activates both STAT3 and STAT5 in humanized mice, suggesting the critical role of STAT3 and STAT5 for normal granulocytic differentiation from hematopoietic stem cells. Therefore, activation of both STAT3 and STAT5 is likely required for granulocytic differentiation. We assumed that STAT3 might already be activated in HT93A, and there might be little margin for additional response in the presence of G-CSF. Similar to our findings, APL cell line UF-1 is resistant to ATRA but differentiation can be induced with ATRA plus G-CSF [29,30]. These studies investigated STAT3 activation, but failed to explain the contribution of STAT3 to G-CSF-induced granulocytic differentiation in combination with ATRA [29,30]. We believe ATRA promotes STAT5 activation by G-CSF, but not STAT3. However, we did not evaluate JAK-related pathways other than JAK-STAT, such as ERK, PI3K, AKT, and MAPK. Other JAK-related signaling pathways might contribute to granulocytic differentiation in HT93A cells.

We could not determine why STAT5 was activated by G-CSF in the presence of ATRA in our study. We hypothesized that ATRA may up-regulate STAT or G-CSF receptor expression; however, our data showed that STAT3 and G-CSF receptor expression were reduced by ATRA treatment. These phenomena may be due to a negative feedback mechanism. Indeed, multiple negative feedback mechanisms such as reduction of the cytokine receptor, the presence of suppressor of cytokine signal proteins, and inhibition of STAT DNA-binding activity has been suggested [13,31]. Although additional studies are needed to resolve the mechanism underlying the combined effect of G-CSF and ATRA, we assume that any factor that would be affected by ATRA treatment may lie between the cascade of G-CSF receptor and STAT activation.

We used the JAK inhibitor ruxolitinib to verify the requirement of JAK for STAT activation. Inhibition of JAK1, JAK2, and JAK3 as indicated by IC_50_ is reported to be 3.3 nM, 2.8 nM, and 428 nM ruxolitinib, respectively [32]. Consistent with these differences in sensitivity, 320 nM ruxolitinib was considered sufficient for inhibition of JAK1 and JAK2, which are critical for G-CSF signaling [32]. Differentiation of HT93A cells treated with ATRA and G-CSF was abrogated by adding ruxolitinib; the JAK inhibitor reduced differentiation to levels obtained with ATRA alone. However, ruxolitinib did not inhibit ATRA-induced differentiation. These results suggest that the major mechanism of G-CSF-induced granulocytic differentiation is activation of the JAK-STAT pathway, although this pathway is not important for ATRA-induced differentiation.

Because other ATRA-sensitive AML cell lines are not capable of responding to G-CSF even in the presence of ATRA, we investigated the specificity of the HT93A response in comparison to NB4 and THP-1. We suggest the difference is attributable to the unique characteristics of HT93A. Compared to NB4 or THP-1, HT93A cells exhibit distinct degrees of differentiation via CD34 positivity, suggesting their immaturity as myeloid-linage progenitor cells. It should be noted that G-CSF specifically affects progenitor cells that correspond to a specific granulocytic differentiation degree as shown by the CD33^+^/CD34^+^ immunophenotype [33]. Interestingly, HT93A cells exhibit a differentiation stage equivalent to that of the progenitor cells, which are the major target of G-CSF [24,34]. The differential response is likely due to the differences in G-CSF receptor expression in these cell lines, in addition to differences in CD34 expression. Indeed, G-CSF receptor expression was repressed in ATRA-treated HT93A cells, as was granulocytic differentiation as indicated by the reduction in CD34 expression. These findings demonstrate the utility of HT93A cells as a model for understanding the effects of G-CSF and ATRA.

## Conclusions

In conclusion, STAT5 activation (not STAT3) is likely essential for G-CSF-induced granulocytic differentiation in HT93A. This unique cell line mimics normal progenitor cells and may become a useful model for analyzing the effects of cytokine-dependent myelopoiesis, proliferation, and differentiation of hematopoietic cells.

## Materials and methods

### Reagents

ATRA was purchased from Sigma (St. Louis, MO), dissolved in ethanol to 2 mM, and stored at −20°C in the dark. The final concentration of ethanol (≤0.05%) did not affect cell viability or differentiation. Recombinant human G-CSF (Filgrastim) was obtained from Kyowa Hakko Kirin Co, Ltd. (Tokyo, Japan), dissolved in phosphate-buffered saline (PBS) to prepare the stock solution, and stored at −20°C. The JAK inhibitor ruxolitinib was purchased from ChemScene (Monmouth Junction, NJ) and dissolved in dimethyl sulfoxide to 10 mM. Primary antibodies, phycoerythrin (PE)-conjugated mouse anti-human CD11b IgG_2α_, fluorescein isothiocyanate (FITC)-conjugated mouse anti-human CD34 IgG_1_, and PE-conjugated mouse anti-human G-CSF receptor IgG_1_ antibody were purchased from Becton-Dickinson (San Jose, CA). Non-binding mouse IgG-PE and IgG-FITC isotype antibodies (Becton-Dickinson) were used as controls. Antibodies for intracellular staining, rabbit anti-STAT3 (#GTX108630), anti-STAT5A (#GTX103750), anti-phosphorylated STAT3 (Y705; #GTX118000), and anti-phosphorylated STAT5 (Y694; #GTX13593) polyclonal antibodies, were purchased from GeneTex (Irvine, CA); anti-STAT5B polyclonal antibody (#bs-4254R) was from Bioss (Woburn, MA). FITC-labeled goat anti-rabbit IgG secondary antibody was obtained from Kirkegaard & Perry Laboratories (Gaithersburg, MD). Non-binding rabbit IgG (GeneTex) was used instead of primary antibody as negative control.

### Cell culture

HT93A, a human APL cell line established from the peripheral blood of a patient with APL [23], was provided by Dr. Kenji Kishi (Shibata Hospital, Shibata, Japan) and Dr. Yuko Sato (National Center for Global Health and Welfare, Japan). HT93A, NB4 (APL), and THP-1 (acute myelomonocytic leukemia) cells were maintained in RPMI-1640 medium (Sigma) supplemented with 10% heat-inactivated fetal bovine serum (Gibco-BRL, Grand Island, NY), 100 U/mL penicillin, and 100 μg/mL streptomycin (Gibco-BRL) at 37°C in a humidified atmosphere (5% CO_2_ in air).

### Growth inhibition

HT93A cell viability was investigated by trypan blue exclusion assay. Cells that stained negative and positive with trypan blue were considered as viable and dead, respectively. Growth inhibition was expressed as a ratio of the number of viable cells in each treatment group to the control group.

### Differentiation marker and G-CSF receptor expression

Differentiation induction was confirmed by surface marker expression. Myeloid maturation was analyzed by FACSCalibur cytometry (Becton-Dickinson) with CD11b, CD34, and G-CSF receptor antibodies as described with minor modifications [24,33,34]. In brief, approximately 1 × 10^6^ cells were washed with PBS containing 2.5% fetal bovine serum and 0.5% NaN_3_ (PBSF) and stained with PE-conjugated mouse anti-human CD11b IgG_2a_, PE-conjugated mouse anti-human G-CSF receptor IgG_1_, and FITC-conjugated mouse anti-human CD34 IgG_1_ for 30 min at 4°C in the dark. Cells were washed twice with PBSF and analyzed by flow cytometry with a minimum acquisition of 10,000 events. Non-binding mouse IgG-PE isotype antibodies or non-binding mouse IgG-FITC isotype antibodies were used as controls.

### Intracellular staining

Flow cytometry analysis was performed to analyze intracellular protein expression after fixation and permeabilization [35,36]. Cells were collected by centrifugation and fixed with 4% formaldehyde for 10 min at 37°C. After fixation, cells were permeabilized by adding ice-cold 100% methanol to a final concentration of 90% and incubated at −20°C for 30 min. After washing twice with PBSF, cells were stained with each primary antibody for 30 min at 4°C, followed by staining with FITC-conjugated secondary antibody for 30 min at 4°C in the dark. Cells were washed twice with PBSF and analyzed by flow cytometry with a minimum acquisition of 10,000 events. Expression of each protein is reported as mean fluorescence intensity (MFI).

### Statistical analysis

Experiments were independently repeated three times, and results shown are the mean ± standard deviation (SD). Data were analyzed using paired Student’s *t*-test, and p < 0.05 was considered statistically significant.
